# ResNetFed: Federated Deep Learning Architecture for Privacy-Preserving Pneumonia Detection from COVID-19 Chest Radiographs

**DOI:** 10.1007/s41666-023-00132-7

**Published:** 2023-06-14

**Authors:** Pascal Riedel, Reinhold von Schwerin, Daniel Schaudt, Alexander Hafner, Christian Späte

**Affiliations:** 1 Institute for Informatics, University of Applied Sciences, Prittwitzstraße 10, Ulm, 89075 Baden-Württemberg Germany; 2 Transferzentrum für Digitalisierung, Analytics & Data Science Ulm (DASU), Ensingerstraße 4, Ulm, 89073 Baden-Württemberg Germany

**Keywords:** Federated learning, Deep learning, ResNet, Medical imaging, COVID-19

## Abstract

Personal health data is subject to privacy regulations, making it challenging to apply centralized data-driven methods in healthcare, where personalized training data is frequently used. Federated Learning (FL) promises to provide a decentralized solution to this problem. In FL, siloed data is used for the model training to ensure data privacy. In this paper, we investigate the viability of the federated approach using the detection of COVID-19 pneumonia as a use case. 1411 individual chest radiographs, sourced from the public data repository COVIDx8 are used. The dataset contains radiographs of 753 normal lung findings and 658 COVID-19 related pneumonias. We partition the data unevenly across five separate data silos in order to reflect a typical FL scenario. For the binary image classification analysis of these radiographs, we propose *ResNetFed*, a pre-trained ResNet50 model modified for federation so that it supports *Differential Privacy*. In addition, we provide a customized FL strategy for the model training with COVID-19 radiographs. The experimental results show that ResNetFed clearly outperforms locally trained ResNet50 models. Due to the uneven distribution of the data in the silos, we observe that the locally trained ResNet50 models perform significantly worse than ResNetFed models (mean accuracies of 63% and 82.82%, respectively). In particular, ResNetFed shows excellent model performance in underpopulated data silos, achieving up to +34.9 percentage points higher accuracy compared to local ResNet50 models. Thus, with ResNetFed, we provide a federated solution that can assist the initial COVID-19 screening in medical centers in a privacy-preserving manner.

## Introduction

Data privacy and regulatory compliance are increasing constraints in the areas of Machine Learning (ML) and Deep Learning (DL) [1–3]. Regulatory frameworks such as the General Data Protection Regulation (GDPR) for Europe [4], the Health Insurance Portability and Accountability Act (HIPAA) for America [5], or the Personal Information Protection Law (PIPL) for China [6] make centralized data-driven analytics while preserving data privacy substantially more challenging. In areas where highly critical data is stored, such as in medical centers, the application of data-driven methods is often not feasible due to these regulations [3, 7]. Failure to comply with data protection regulations can result in heavy fines [4, 8]. For example, a data protection breach in which personal data is leaked outside the company is fined with up to 20 million euros or 4% of total revenue in the fiscal year through the application of Article 83 in the GDPR [9]. From an economic and legal perspective, data protection is therefore an important prerequisite for the use of ML and DL with personal data [8]. This is especially important in healthcare [1, 10], where the question arises of how sensitive personal, cross-site information can be analyzed using data-driven techniques while taking data privacy into account.

Federated Learning (FL) addresses this question [10]. Already introduced as an early novel concept by Google in 2017 [11, 12], FL is a decentralized learning architecture in which different data silos holding sensitive data jointly solve a ML or DL task without the data itself having to be exchanged. A data silo refers to a repository that stores sensitive training data, such as thin nodes, fat clients, or embedded devices [11]. The model training is performed locally in each silo, without moving any training data between the silos [12]. By regularly exchanging model updates between the data silos and a central coordinator server, a global single learning model can be inferred [11]. FL is sometimes referred to as *federated optimization* to imply its similarity and contrast with distributed ML optimization [11, 12]. Although this term might be technically more accurate in describing the task itself, we use the more general term FL in this paper.

This paper looks at diagnosing COVID-19 based on radiographs as a use case. Despite proven vaccines and extensive awareness, numerous people are infected with COVID-19 every day, while the risk of severe disease progression remains and those who have recovered may suffer from protracted COVID-19 symptoms [13–15]. For hospitalized COVID-19 patients, chest radiographs are taken for diagnosis [16]. These images assist physicians to identify and classify the severity of pneumonia, which is usually associated with a severe course of COVID-19 [13, 16]. Our work aims to successfully distinguish pathogenic findings from normal findings using radiographs of the lungs in an automated fashion *that complies with data protection regulations*. For this binary image classification task, we investigate the application of a federated approach in the restrictive healthcare environment where data privacy compliance is crucial. Assuming that we use an FL approach which preserves Differential Privacy (DP; S. Section 3.2), the questions we want to answer are:

1. Can the FL approach provide results comparable to a centralized model?
2. Is there a benefit of using FL compared with locally trained models?

We address these questions and simulate an FL system consisting of five data silos in which the lung radiographs are distributed differently to evaluate the impact of unfavorable data distributions on the global learning model. For the COVID-19 detection we use a pre-trained ResNet50 model. Since ResNet models with DP cannot be federated by default, we propose *ResNetFed* which is a modified federated pre-trained ResNet50 model (S. Section 3.2 for details) that enables the use of DP in an FL system. Additionally, we present a customized FL strategy for the training of ResNetFed.

The remaining part of the paper is organized as follows: Section 2 describes works closely related to this paper. An overview of FL, a description of the dataset used, the proposed modification of the ResNet architecture and a federated learning strategy are provided in Section 3. In Section 4, we elaborate on experiments and compare ResNetFed models with local and centralized standard ResNet50 models. The paper finishes with a general conclusion in Section 5.

## Related Work

In the past, there was a lot of research focused on the detection of pneumonia on radiographs by applying centralized DL algorithms. Sharma et al. [17] evaluated different deep Convolutional Neural Networks (CNNs) with augmented images. They achieved a test accuracy of 90% by using dropout layers and a set of different augmented image techniques. On the other hand, [18] examined various pre-trained CNN models to provide a reliable data-driven COVID-19 screening. Wang et al. [19] achieved a true positive rate of 91% using COVID-Net, a customized DL architecture for COVID-19 detection. [20] classified chest radiographs into normal and positive findings using hierarchical CNNs. Chowdhury et al. [21] used pre-trained CNNs for COVID-19 detection with chest radiographs. Here, the authors consistently achieved high test accuracy values of more than 97%. They applied different model architectures for this purpose. Interestingly, ResNet18 and ResNet101 models were also tested, with the ResNet18 model generalizing slightly better than the deeper ResNet101 model [21]. A ResNet50 model, on the other hand, was not included in the model pool of [21]. Nevertheless, ResNet50 seems to be an appropriate model for the initial diagnosis of COVID-19 on radiographs because it is smaller, faster to train, and less likely to overfit than a ResNet101 and therefore forms a good candidate between neural networks that are too shallow or too deep [21, 22]. In addition, the works of [21, 23–26] showed that pre-trained deeper or shallow ResNet models do not necessarily perform better than ResNet50 models on classification problems. Srivastava et al. [27] introduced CoviXNet a customized CNN model consisting of 15 layers that exhibits a cross-validation accuracy of 99.47% for the COVID-19 detection with radiographs. Srivastava et al. [28] proposed a domain extended transfer learning ensemble method and compared it with an ensemble method based on Condorecet’s Jury Theorem. The author’s best ensemble model achieved an accuracy of 98.22%. Srivastava et al. [29] also used an ensemble model based on Condorect’s Jury Theorem, which comprised six CNNs as base learners and used a heuristic optimization algorithm to determine the optimal weights. In that work, the author’s best performing model attained a test accuracy of 99.78% for colorectal cancer classification using histopathological images. An analysis of different DL models conducted by [30] demonstrated a pre-trained InceptionV3 model that exhibits a promising test accuracy of 99.78% for a binary classification with COVID-19 radiographs. All this research shows that a pre-trained CNN is a versatile tool for image classification of pathogenic radiographs. It is worth mentioning that some researchers used alternative model types such as long-short term memory, variational autoencoders or k-nearest neighbors for classification tasks with radiographs [31–33]. However, in general, these models have not shown improved performance compared to CNNs [17–21, 31].

Centralized ML models, however, face a significant challenge as they require mandatory access to centralized data sets. Sensitive data such as radiographs are personal and therefore subject to data protection laws [5, 6]. As mentioned at the beginning, recourse claims can therefore arise if the data is leaked [9]. Especially in the healthcare industry, data protection of patient data must be strictly observed [4, 10]. The European Union Agency for Cybersecurity (ENISA) defined some guidelines to facilitate the privacy-compliant handling of sensitive data [34]. Most of the ENISA principles can automatically be fulfilled when FL is used instead of a centralized learning approach. By keeping the sensitive data in the individual data silos (e.g. image databases in hospitals) and not moving them, the privacy aspect for data-driven analyses in healthcare is respected (privacy by design). This is also confirmed by the work of [24] who classified pathogenic lung radiographs from COVID-19 patients using federated pre-trained CNN models. They used a federated VGG16 as well as a federated ResNet50 model and achieved comparable accuracy values to non-federated models. The federated ResNet50 model achieved a test accuracy of 95.4% without augmented images. However, there are several aspects in the work of [24] that motivate further research:

- There is no indication that privacy mechanisms were used for federating the models, which might otherwise affect the model quality.
- It is not clear whether architectural changes were required to federate a ResNet50 model.
- The dataset was rather scarce with 108 radiographs (20% of which were test images) belonging to 76 individuals.

In this paper, we address these aspects and demonstrate that our proposed FL strategy enables a reliable and privacy-compliant COVID-19 screening with chest radiographs. Our paper shows that pre-trained ResNet50 models can be securely federated under certain architectural modifications and aspects. Based on the research literature, federated pre-trained ResNet50 models have rarely been studied in more detail with a larger dataset and silos, which motivates our work. Although there are superior pre-trained CNNs, we limit the model selection to ResNet50 because we are more interested in leveraging FL with a pre-trained CNN with DP and how it performs in direct comparison with a local non-federated model. Additionally, a silo-based comparison between the federated solution and a centralized learning strategy is performed to understand the ramifications of the data distributions between the silos.

## Federated Optimization with Radiographs

**Fig. 1:**
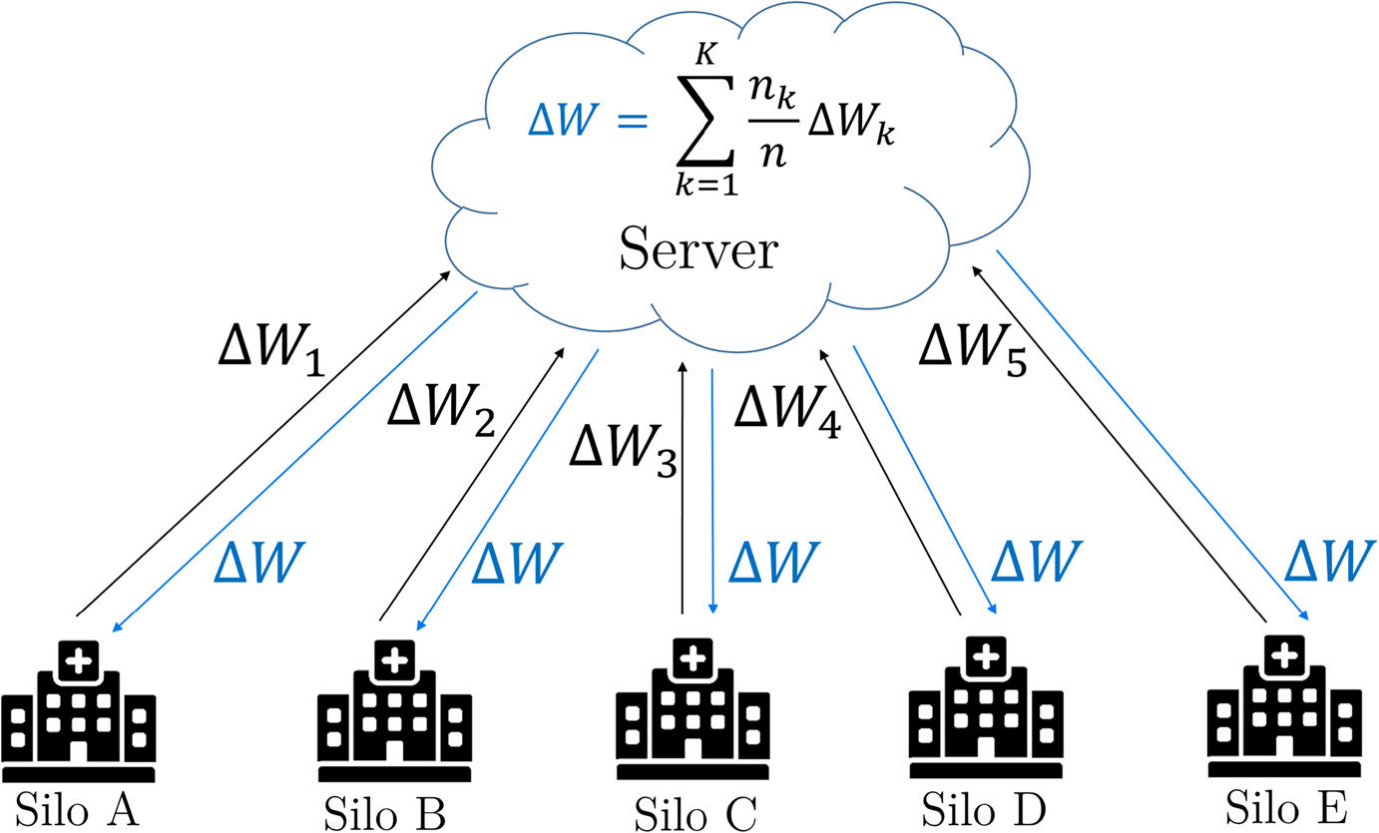
Federated learning architecture for radiograph-based COVID-19 detection with *K* silos, *n* radiographs, and weighted FedAvg. Each silo exemplifies a unique hospital. The blue lines and $\Delta W$ represent the averaged model weights returned to *K* silos

A federated learning system always consists of at least one server and *K* data silos [11, 12]. In this decentralized learning architecture, the server initializes a global model $\Delta W$ which is sent to *K* silos. Subsequently, the models $W_k$ in each of the silos are trained locally with the silo data. After the local training, the updated models $\Delta W_k$ are sent back to the server. In this configuration the server works as a coordinator and aggregates these models, creating a new global model $\Delta W$. This new global model is again distributed by the server to the *K* silos simultaneously. Such a training round is called a *communication round* in the literature [11, 12, 24, 35]. However, we use the alternative term *federated training round* in this paper, which is more precise. The federated training continues until *t* federated rounds are completed and the global model converges [12]. As an aggregation strategy, [11] proposed the *FederatedAveraging* (FedAvg) algorithm, where a weighted arithmetic mean of $\Delta W_k$ is formed. This ensures that each silo contributes to the global model based on the amount of training data resting in the silos. Figure 1 shows our federated learning architecture for the COVID-19 screening task. For the image classification task, all available radiographs from *K* silos in *t* federated training rounds are considered. The model training and the exchange of model updates, as shown in Fig. 1, are processed in parallel [11]. The server waits until *K* silos have sent a model update before aggregating a new global model [12]. Therefore, the number of silos must be set in advance.

In federated optimization, it is assumed that *n* data are resting among *K* silos [12]. Then, the aggregation strategy with weighted FedAvg represents a distributed optimization task [12, 24] where the objective is to minimize the global loss function:

1 $$\begin{aligned} \min _{w \in \mathbb {R}^d} \; f(w) \quad \textrm{where} \quad f(w)&=\sum _{k=1}^K \frac{n_k}{n}F_k(w)\\ \textrm{with} \quad F_k(w)&= \frac{1}{n_k}\sum _{i=1}^{n_k}f_i(w) \nonumber \end{aligned}$$

where *w* is a weight vector from a neural network, $f_i(w)$ is a loss function, *K* is the number of participating silos and $n_k$ is the number of data per silo. Thus, $F_k(w)$ describes a local loss function $f_i(w)$ for a prediction with weight vector *w* at silo *k*. Then, the aggregated and averaged loss values over all silos are represented by *f*(*w*). This means, the optimization task (s. Equation 1) includes the minimization of a convex local loss function of the neural network at each data silo assuming synchronous updating at the server side (s. Figure 1). As pointed out by [12] for non-convex loss functions, averaging model weights in a parameter space might yield an arbitrarily bad model. To distinguish penumonia from normal lung findings, our proposed federated model uses the binary Cross-Entropy (CE) loss function $CE(f_s(x), Y)$ [36]. Here $f_s(x)$ refers to the softmax function for logit *x* and *Y* refers to the possible output labels.

### Data

**Fig. 2:**
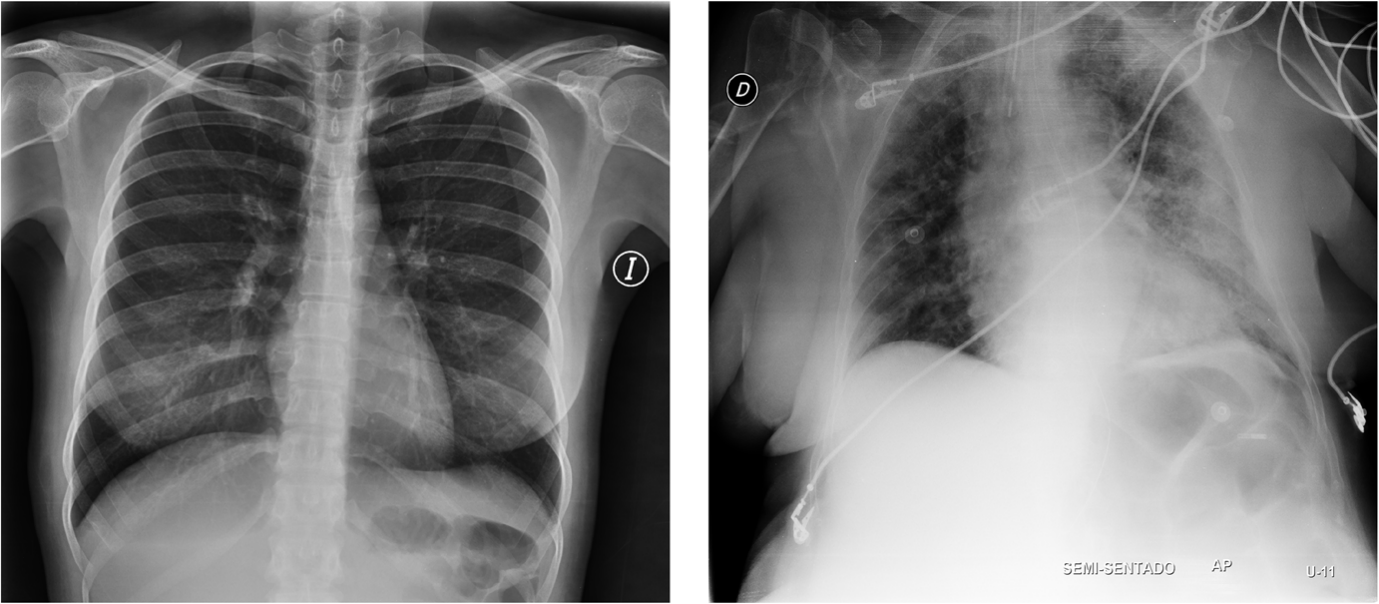
Resized chest radiographs of two patients from COVIDx8 [37]. Left side shows normal finding (COVID-19 negative) and right side shows severe pneumonia (COVID-19 positive)

For the federated-driven COVID-19 analysis we use 1411 different chest radiographs from the open access benchmark dataset, i.e., COVIDx8 [37]. The images in this data repository were provided by a consortium of medical organizations and used for the development of COVID-Net [19]. Chest radiographs of COVID-19 patients show findings such as milk glass opacities in the absence of pleural effusion and can therefore be used as a tool for initial COVID-19 diagnosis [38]. Lung findings range from normal in the early stages of the disease to unilateral or bilateral lung opacities, sometimes with basilar and striking peripheral distribution [39]. However, these radiographs present an optimization problem from a data science perspective. Thoracic radiographs often have low contrast and are difficult to distinguish as a non-expert. Figure 2 shows a pair of images that illustrate this problem.

Training data that are difficult to differentiate can also lead to poor generalizability and thus increase the likelihood of misclassifications [40]. By performing the same data preparation steps for the radiographs in all data silos using a sufficiently large data set, this problem can be minimized. The radiographs are scaled down to an image size of $224 \times 224$ and normalized with the mean and standard deviation of images from the ImageNet [41] dataset. Although not all COVIDx8 images are isotropic and may introduce distortions into the data, we still use a symmetric image size of $224 \times 224$. These standard pixel sizes are well well covered by the CNN’s filter. The downsized images do not appear overly stretched, and the same pixel size was also used in other similar works [22, 24].

To investigate the application of FL with unevenly distributed data between the silos, without the bias of augmented data, we decided to exclude augmentation. Although augmentation could increase the generalizability of the model, it is not relevant for the comparison between federated and local models. Furthermore, the inclusion of augmented radiographs would require a more complex data pipeline, increase training times, and cause stochastic variations between federated and local models.

### Proposed Model Architecture

The ResNet50 model proposed for the COVID-19 detection task was pre-trained on the ImageNet dataset consisting of 14 million images divided into 1000 classes [41, 42]. A class for lung thoracic radiographs was not included. Nevertheless, numerous papers confirmed that the pre-trained model is able to classify COVID-19 radiographs through the concept of transfer learning [18, 20, 31, 41, 42].

For privacy-compliant COVID-19 screening, a security mechanism as studied by [43] should also be implemented to make the FL system more robust against data exposures. Thus, for our proposed model architecture we implement DP which guarantees privacy during the aggregation step in FedAvg [43, 44]. DP can be defined formally as follows:

2 $$\begin{aligned} Pr\left[ M(D) \in Z \right] \le e^\epsilon Pr\left[ M(\bar{D}) \in Z \right] + \delta , \end{aligned}$$

where *Pr* indicates privacy, *M* is the federated model, *D* and $\bar{D}$ are two random neighboring datasets that have only one single different sample, *Z* denotes a set of outputs and $\epsilon$ specifies the privacy budget [43, 45]. Thus, after applying DP, *M* is $\epsilon$-differentially private [46]. The additive term $\delta$ (s. Equation 2) denotes a tiny failure probability that our federated model admits a privacy-violating configuration and serves as a bound [43]. Our proposed model uses DP with Stochastic Gradient Descent (SGD) which computes a noisy sum of gradients consisting of random samples given a sampling probability, clipped gradients, and a Gaussian noise which is added to the sum [43, 45, 46]. Adaptive optimizers for DP are not implemented by default and are not supported by the FL framework [43, 47]. DP-SGD returns an anonymized federated model so that observations of our model will not reveal any information about the silo’s dataset on which the model was trained [44, 46].

Unfortunately, ResNet architectures have batch normalization layers [42] which cannot work with DP-SGD as they violate the assumption DP-SGD uses to guarantee privacy [43, 46]. Namely, DP-SGD assumes that there is no dependency between samples in a batch [46]. However, the batch normalization function used in ResNet models calculates mean and variance *across* batches and thus creates a dependency between samples in different batches which leads to a privacy violation so that the model cannot be federated with FedAvg and DP-SGD [44, 48, 49]. Another issue can occur during federated training when the momentum value for batch normalization is set to zero [49] leading to float values instead of integer values due to the averaged aggregation. Batch normalization layers are designed for centralized learning and not for federated settings. Therefore, these layers should be disabled to enable FL with ResNet models.

**Fig. 3:**
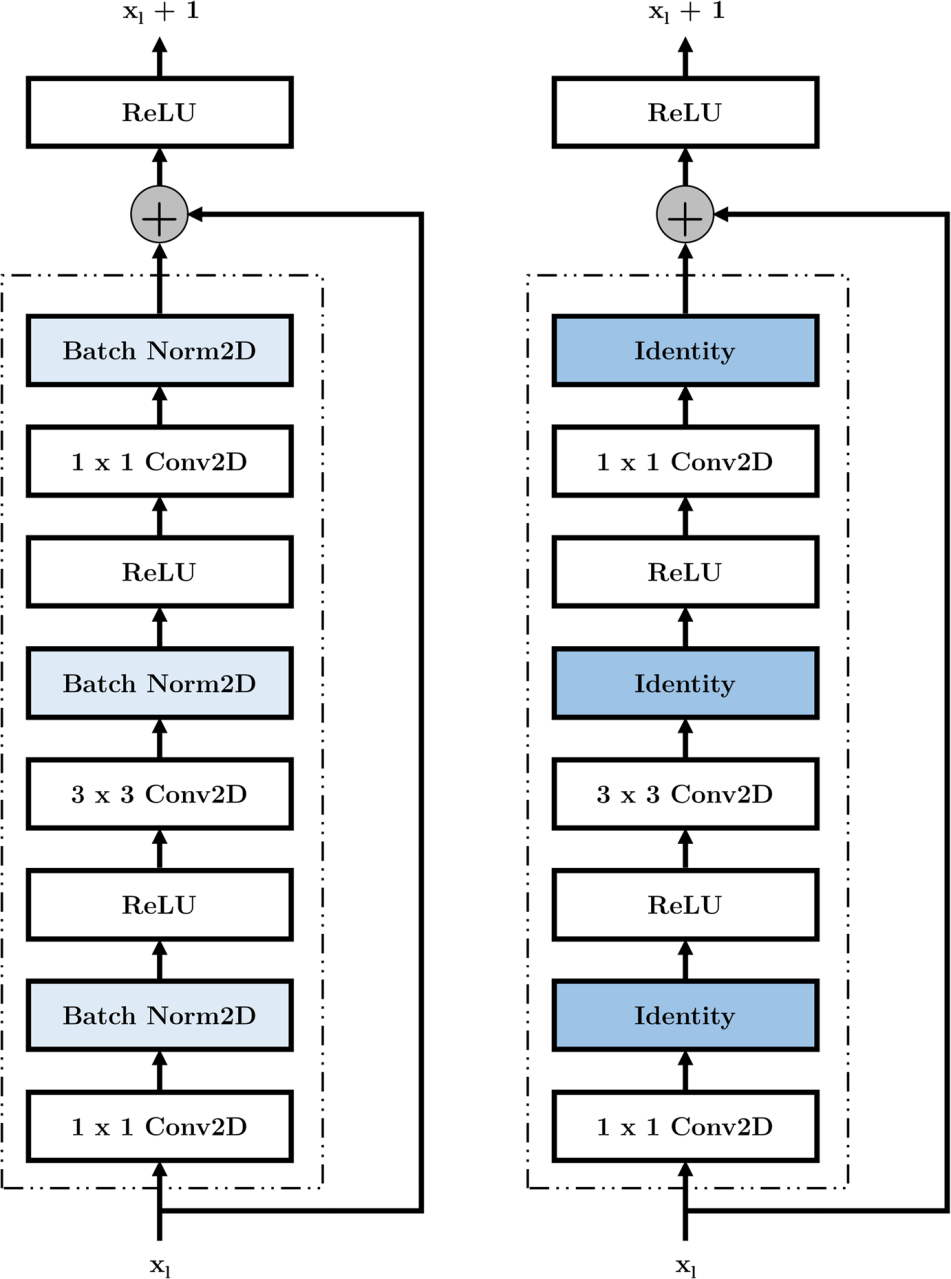
Identity blocks of ResNet50 architecture for input layer $x_l$ with identity shortcuts. Left side shows original block with two-dimensional pre-trained batch normalization layers in light blue [42], right side shows modified block where the batch normalization layers are replaced by identity layers in dark blue

Motivated by the principle of skip connections in the original ResNet architecture [42], we replace all batch normalization layers with identity layers. With these identity layers the inputs are passed from the batch normalization layers directly as output [50]. We call the model ResNetFed because the architectural changes enable federation with DP-SGD. Figure 3 shows the necessary architectural changes to enable a privacy-compliant federated ResNet50 model.

ResNetFed takes as input chest radiographs (s. Figure 2) with an image size of $224\times 224$ and outputs two probability values indicating the presence or absence of pneumonia. The only disadvantage of the newly proposed model architecture compared to the legacy one might be the lost pre-trained weights of the batch normalization layers, which may lead to a slower convergence. However, for FL, this should still be an acceptable trade-off, since most of the learned information is contained in the pre-trained weights of the convolutional layers. In addition, the proposed architectural modifications for ResNetFed can also be applied to other ResNet models, such as ResNet34 or ResNet152.

### Proposed Learning Strategy

For the COVID-19 classification task, ResNetFed uses weighted FedAvg. Here, the model is initialized with pre-trained weights $w_0$ from the convolutional layers. As a fitting learning rate $\eta = 5e-4$ and a mini-batch size of $b = 6$ is used. For the gradient optimization, we use SGD with momentum $\beta = 0.1$. For $\epsilon$-DP it is required to use a value from the range 0 to 1. An acceptable one for $\epsilon$-DP seems to be 0.4. Larger values may lead to potentially better peformance but also less privacy protection. We set an upper bound of 7 for clipping the gradients [51]. This helps the FL system to determine how much noise needs to be added to guarantee privacy where the threshold value should scale along with the noises added in DP. This configuration setup is shared on *K* silos. Afterwards, the federated training process is divided into *T* training rounds, where each training round $t_i \in \{1,...,\text {T}\}$ consists of the following four steps:

Step 1. A ResNetFed model with initial weights $w_{0}$ is distributed to $k=5$ silos and trained with local radiographs $P_k$. For validation, each silo contains a separate test set.
Step 2. After a local training epoch $k_i \in \{1,...,\text {E}\}$ has passed with DP-SGD, a weight update $\Delta W_k$ is sent to the server.
Step 3. The server synchronizes and aggregates all weight updates from *K* silos with weighted FedAvg. Then, the server distributes the updated model weights back to *K* silos. A new federated round *t* is started.
Step 4. After $t_i=T=100$, the federated training is terminated and the global model $\Delta W$ is exported for further evaluation.

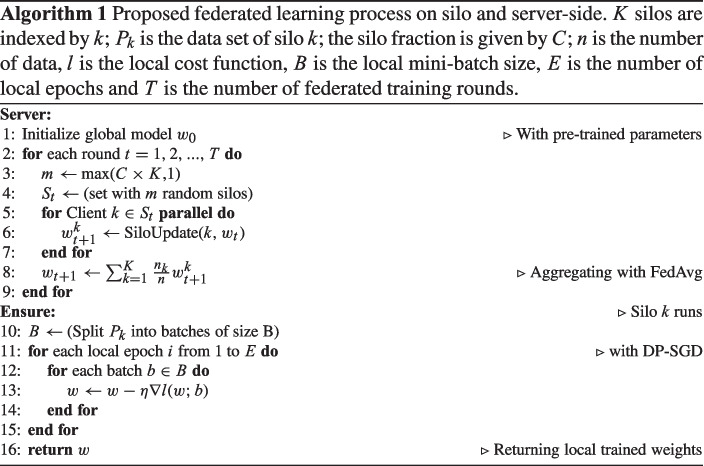


From Step 2, it can be deduced that $E = T$ is true for the proposed learning strategy. More training epochs would lead to a tremendous training workload, since the total number of local training epochs of the network is determined by $E \times T \times K$. We set $t=100$ to provide enough training rounds for finding a stable model configuration with a high prediction power. Algorithm 1 lists the details of the proposed federated learning process between silo and server. It shows that the federated training process is started by the server by distributing a global model to a subset of silos [12, 24, 52]. The fraction of participating silos in *t* rounds can be specified by the parameter *C* [52, 53]. For using all available silos, we set $C=1$. The model training is performed in parallel in each silo [11, 35, 53] by *SiloUpdate*(*k*, $w_t$). Within silo *k*, the model $w_t$ is locally trained on data $P_k$ with DP-SGD. The central server waits until *K* silos have completed their training and returned their anonymized weight updates [11].

Subsequently, *K* updates are aggregated with weighted FedAvg and a new round *t* with the updated model $\Delta W$ is started.

## Experimental Results & Discussion

### Experimental Setup

**Fig. 4:**
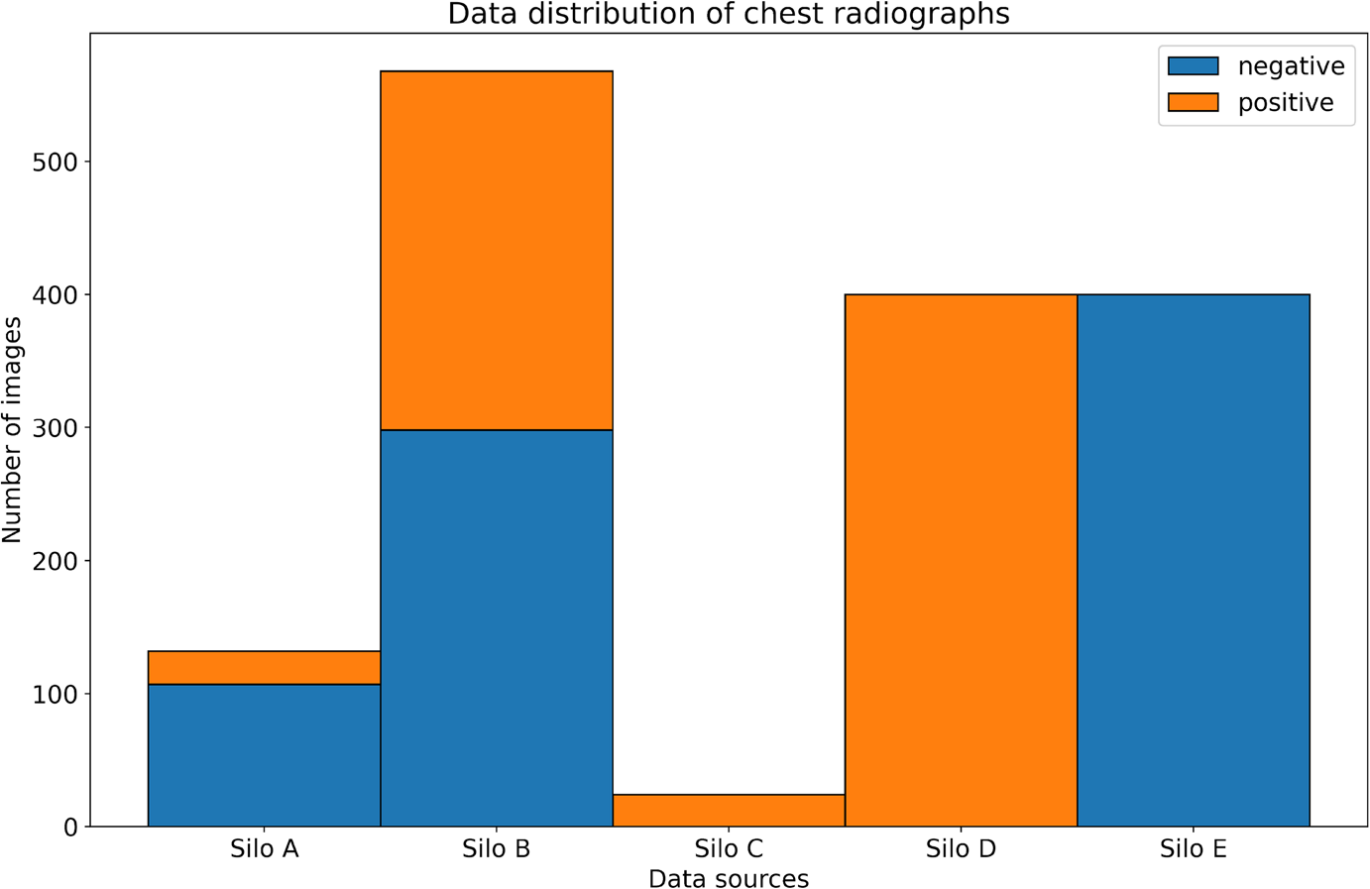
Distribution of 1411 radiographs to silos A - E with two labels (negative findings / positive findings)

For our experiments, we distribute $n=1411$ radiographs differently across $K=5$ separate silos. We use an inhomogeneous data distribution because distributed real-world data is often imbalanced, skewed and poorly distributed as well [54]. Thus, Fig. 4 represents the experimental data distribution for each silo *k* for the experiment setting.

Here silos C - E are special cases because they each have only one label and silo C has the least data compared to all other silos. This should help to evaluate whether silos C - E can benefit from the training data of silos A and B through the application of FL. Silos A and B contain data from both labels, with silo A being severely imbalanced. The data distribution shown in Fig. 4 exhibits a positive skewness, which has the potential to result in inaccurate estimations of the mean and standard deviation of the data. This could have a detrimental effect on the model quality. We use a suboptimal data distribution to investigate how FL handles poorly shaped training data and whether it increases the federated model performance compared to locally trained models.

For the federated training, all data $P_k$ in *K* silos is used for training and 20% additional data form the test sets (holdout testing), with a shuffle split applied to $P_k$. Due to the minority number of samples in silo C we use the same test set in C - E. As demonstrated in Fig. 4 it is clear, that the experimental data is heavily *Non-Independent and Identically Distributed* (Non-IID). The model accuracy during FL with weighted FedAvg on highly biased Non-IID data is usually lower than for a centralized model, even for convex optimization cases [55]. The weights and biases during training diverge more in Non-IID settings than in IID settings leading to a reduced generalizability of the global model [56, 57]. We attenuate the Non-IID data problem by providing enough training data ($n=1411$) on a small set of silos ($K=5$) and applying SGD with a momentum value of $\beta = 0.1$. Using the default value of $\beta =0.9$ for our dataset results in vanishing gradients during federated training. One reason for this could be that SGD momentum is quite sensitive in FL settings, since it only tracks local weight updates on silo *k* and the same $\beta$ is used for all silos, regardless of their data distribution. Tests showed that in silos C - E the vanishing gradients already occurred after the first federated training round when the default value for SGD momentum is used. Then, the vanishing gradient updates were aggregated and distributed to *K* silos, resulting in *K* silos with vanishing gradients. There is an experimental strategy to use an aggregated averaged global momentum that keeps the gradient history of each silo [58]. However, in most practical scenarios the data on the individual silos are heavily Non-IID, thus aggregating one global model may lead to suboptimal local model updates on silo-side during federated training [55–57].

The silo-side training is run locally on a dedicated GPU server with two RTX 3090 GPUs with CUDA acceleration in our institution. A separate Docker container instance is created for each data silo, which is intended to simulate the isolation and separability of silos A - E on one device. As a Python Framework, Integrate.ai which provides an end-to-end SDK for creating custom federated applications is used [47]. The coordinator server which aggregates the anonymized model updates is located at Integrate.ai in Toronto, Canada.

### Model Comparison

**Fig. 5:**
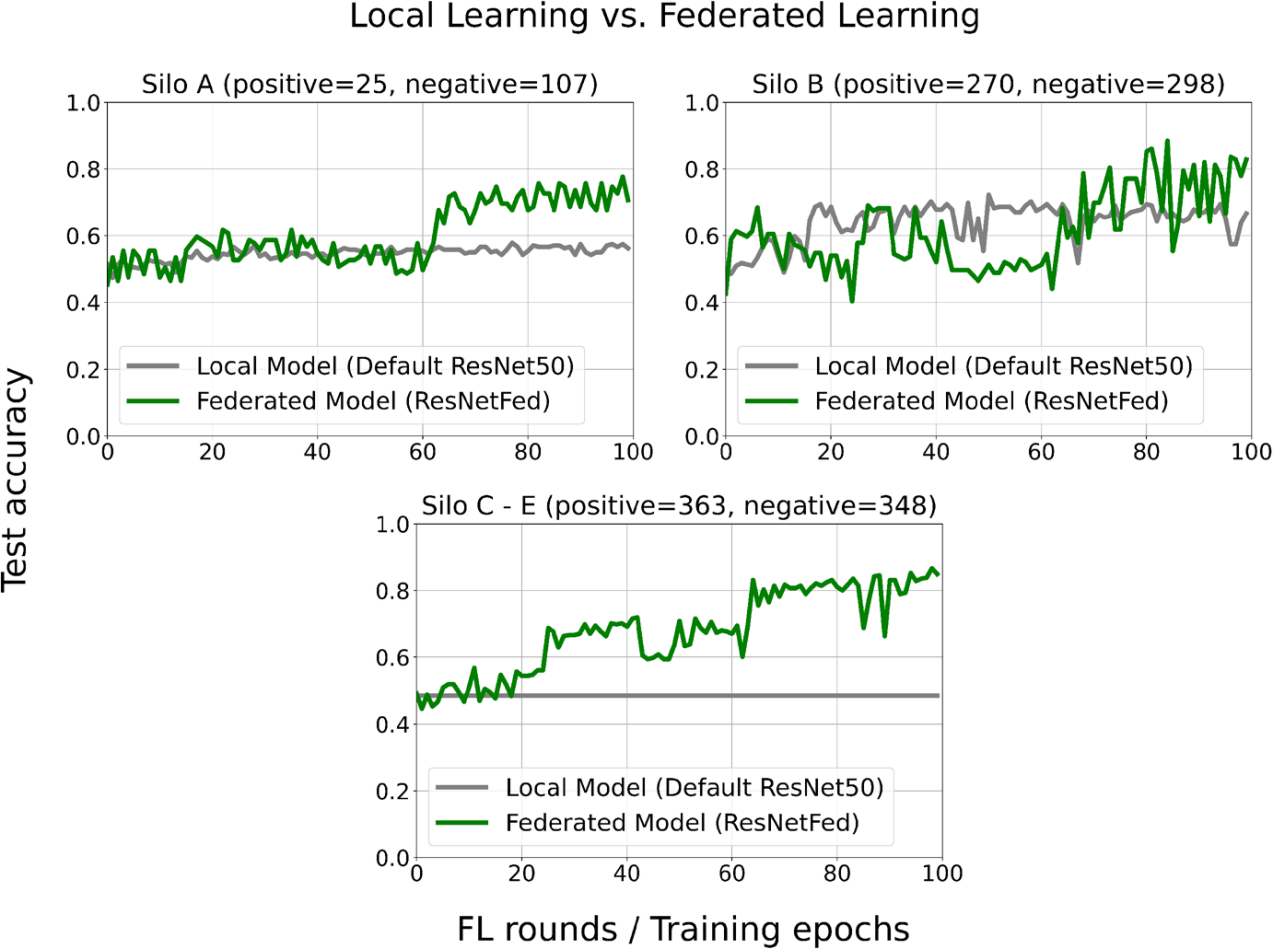
Comparison of pre-trained default local ResNet50 and ResNetFed model training rounds in the silos A - E. Distribution of training data is given in parentheses

The federated learning strategy is compared with a local learning strategy in which a standard pre-trained ResNet50 model is trained in silos A - E with the respective silo data and a centralized model as baseline. Comparing the two approaches should increase the interpretability of FL-driven systems and thus contributes to the reliability of FL. In order to evaluate the quality of the ResNet50 models, we report accuracy scores on testing data after each federated training round *t*. By training exactly one local epoch $k_i$ in each federated round *t*, we can compare the locally trained models with the federated model in each silo *k*. We also include the hypothetical centralized test case where the data from all 5 silos is stored in one location. This should demonstrate how the model quality is affected by the number of available silos and their data distributions. Figure 5 shows the experimental results of comparing our proposed federated learning strategy with the modified ResNet50 model (ResNetFed) and a centralized approach with an unmodified ResNet50 model.

**Fig. 6:**
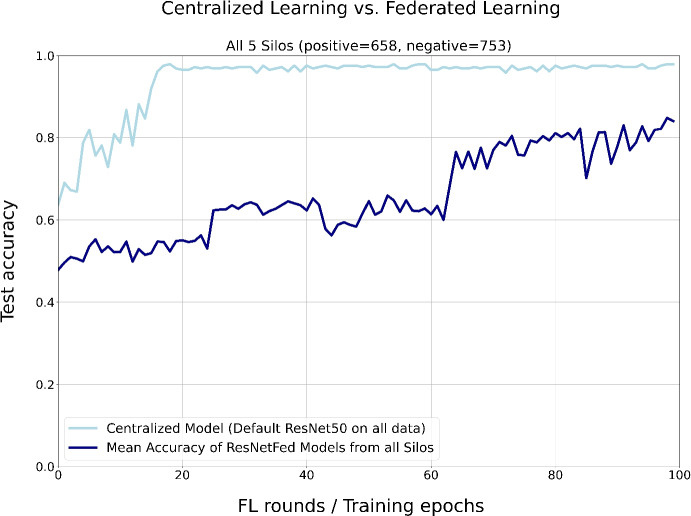
Comparison of test accuracies of a pre-trained centralized ResNet50 with data from silos A - E in light blue and the mean test accuracies from the ResNetFed models of silos A - E in navy blue. Distribution of training data is given in parentheses

As can be noted from the comparison, silos A and B seem to have difficulties with their local prediction models in classifying new unseen data. After 100 training epochs, the local model in silo B achieves a test accuracy of about 63%, but does not achieve a significant improvement compared to its first 20 training epochs. This can be justified with less training data, so the local models in silos A and B are likely to overfit and thus the decision of whether a radiograph is pathogenic or normal is rather random.

For the FL models, on the other hand, silo A and silo B show stagnation in training performance only in the first 62 federated rounds. From the 63rd federated training round on, the federated ResNet50 models in A and B have a higher prediction power than the local counterparts. For example, at silo B, the ResNetFed achieves a test accuracy of over 82.82% after $t=100$, which is a 16 percentage points higher prediction accuracy compared to the silo’s local model. In the silos C - E, the models are identical, with the local models having a prediction accuracy of 50% because they are each trained with one class but evaluated with both labels from the same test set (s. Figure 5). Here, ResNetFed achieves up to 34.9 percentage points higher test accuracy compared to the local models.

Interestingly, in all silos the federated model shows an improvement in prediction after $t=25$, then stagnates slightly and improves again after $t=63$. However, the experiments indicate that the total number of training data and their distribution considerably affect the model quality in FL. It is also noticeable that both models oscillate more strongly in silo B than in the other silos. One reason for this could be the larger amount of data and its data quality in silo B. Additionally, any radiographs with only mild pneumonia findings resemble radiographs from normal healthy lungs, which may reduce the generalization ability of the models. Another cause for this could be the globally set learning rate. Since the same hyperparameters in each silo are used, they could influence each local model differently based on silo’s data distribution. This may cause the models to overfit slightly. It is also interesting to observe, that in silo B the oscillation effect is more prominent in the federated model than in its local model. This could also imply that federated models are more instable and responsive to gradient changes in the direction of the local minima than in local models trained with only local data.

In the experiment with all 5 silos demonstrated in Fig. 6, the ResNet50 baseline model in the centralized setting achieves a higher prediction accuracy significantly faster than ResNetFed. Due to the 1411 radiographs, a sufficient amount of data is available for solving a binary classification problem from which the centralized trained model benefits. In contrast, the federated model requires much longer to attain a decent prediction power because of the Non-IID shaped data in silos A - E. Here, it is worth mentioning that a federated model can never perform better than a centralized model when exactly the same shape of data is used [11]. In addition, ResNetFed has no pre-trained batch normalization layers, which is another reason for lower convergence speed. Nevertheless, the FL model reaches an acceptable prediction score of about 82.43% at the end of training. Moreover, it is only through the use of FL possible to add any number of silos (e.g. additional hospitals) in the FL system in a privacy-compliant manner, thus significantly increasing the amount of sensitive data available for training. For example, [59] are trying to be the first institution to establish a federated global network of clinical genomic data for the reliable detection of autism.

Table 1 summarizes the comparison of the three learning settings: centralized learning (as a baseline), local learning and FL. As can be seen from this, all local ResNet50 models perform significantly worse than ResNetFed. It also shows in general that ResNetFed with our proposed FL strategy trains a global distributed model that is always outperforming single local models. However, the baseline classifier could not be entirely reached with ResNetFed in terms of model generalizability. But, the centralized model with data from all silos is not privacy-compliant and thus is not viable in practice, as usually for externals there is no permitted access to the radiographs by regulations (e.g. GDPR) or they are located across sites in different medical centers, where the data acquisition process would entail an enormous effort.

**Table 1:** Model evaluation for ResNetFed and ResNet50 in centralized (baseline), local and FL settings

	Silos A – E		Silo A		Silo B		Silos C - E	
Learning Settings / Metrics	Baseline	Federated	Local	Federated	Local	Federated	Local	Federated
Test Accuracy	**98%**	(mean) 82.43%	56.23%	**70.7%**	66.67%	**82.8%**	50%	**84.9%**
Margin Score	$-15.57$		+14.47		+16.13		**+34.9**	

Test accuracy scores are from last training round ($t=e=100$) and the difference is denoted as a margin score in percentage points

Values in bold shows the increased model performances

### Result Summary

Based on our experimental results, the questions posed at the beginning of this paper can be answered as follows:

1. Can the FL approach provide results comparable to a centralized model?

**Answer:** The ResNetFed model trained with the proposed federated strategy can achieve acceptable results compared with a purely centrally trained ResNet50 baseline model, when enough federated rounds are used, even in case of significant skew in the data. The higher number of rounds necessary are due to the fact that the search for a suitable local minimum takes longer and oscillates more in FL than in centralized learning, and is also affected by the number of distributed silos and their data distributions.

2. Is there a benefit of using FL compared with locally trained models?

**Answer:** When compared with local ResNet50 models of the individual silos, the ResNetFed model performs significantly better (up to 34.9 percentage points improvement in accuracy, s. Table 1) and achieves an improved data privacy due to the successful integration of DP in the proposed federated strategy. This makes the application of FL with DP particularly suitable for organizations that have to work with sensitive data, such as medical centers.

However, the federated ResNet50 model of [24] still generalizes faster and also achieves a higher model performance, that can be explained mainly by a smaller number of radiographs, more uniform distribution of training data, and fewer silos. In turn, our paper shows that sensitive image data such as COVID-19 radiographs can be analyzed and evaluated using the proposed privacy-compliant ResNetFed. The experiments showed that isolated and separated silos can successfully distinguish both labels on unseen radiographs by ResNetFed, without both classes being represented in the training data of the respective silos (s. Table 1). This means that silos with few data and only one label can benefit from the training data of a heavy loaded silo with a high degree of balancing. Thus, the total amount of data of all silos for solving a collaborative analysis needs to be considered when using FL. In addition, our work implies that FL is suitable for many small silos, as long as sufficiently large silos exist. This opens up further use cases that can justify the use of FL apart from the data privacy aspect. At the same time, there is no need to access the raw data of the silos from outside at any time, which again underlines the privacy affinity of FL and enables data-driven analyses for sensitive data. The proposed federated learning strategy (s. Algorithm 1) can also be generally applied to other use cases where high data privacy is required and is not limited to healthcare. Additionally, the architecture modifications for ResNetFed (s. Figure 3) can be utilized for any other ResNet models such as ResNet18 or Resnet101 for federation with DP-SGD, even if projection shortcuts are used instead of identity shortcuts.

### Open Challenges

There are several unresolved challenges in FL that need to be addressed through further active research. One of the most pressing challenges is the requirement for access to sensitive training data. The accessibility of sensitive data is a pre-requisite for conducting exploratory data analysis. Presently, there exist no established methods to facilitate access to siloed data by data scientists or data engineers without revealing the confidential identity of the data in question.

As our experimental results show, imbalanced Non-IID data is a further challenge in FL. Although there have been several research efforts to address this issue [12, 56, 60], poorly distributed data continues to have a detrimental effect on the global model quality, presenting a fundamental problem in FL settings.

In our study, we encountered two additional challenges in the implementation of our FL system: hyperparameter optimization and federating pre-trained DL models. Utilizing the same hyperparameters from centralized model pipelines in a federated setting with highly Non-IID data can result in suboptimal model convergence or even cause training to fail, necessitating careful hyperparameter tuning. For federating pre-trained DL models, modifications to the architecture may be necessary, especially if the layers were designed for centralized learning and do not take privacy mechanisms into account. Despite receiving limited attention in previous research, these two challenges are crucial for maintaining the interpretability and reliability of a FL-driven system.

The studies conducted in [11, 35] provides a comprehensive overview of additional challenges in FL, such as increased communication costs in the underlying infrastructure and the situation where the number of silos surpasses the available data. Despite these challenges, they do not present a major hindrance to the detection of COVID-19 in radiographs.

## Conclusion

In this paper, we demonstrated that the use of ResNetFed to detect COVID-19 disease with pathogenic radiographs is privacy-compliant and that it can differentiate cases of pneumonia from normal findings. Modifications to the deep learning model were required for a privacy-compliant federation because, by default, ResNet50 batch normalization layers with differential privacy cannot be federated. However, using ResNetFed, i.e., by replacing these layers with identity layers, the ResNet50 model can be federated with weighted FedAvg. We also identified the federated optimization problem for chest radiographs and described a solution strategy for it. Our proposed federated learning strategy addresses the high privacy requirements of patient data in hospitals and provides a comparatively cost-effective support framework for the initial diagnosis of COVID-19-related pneumonia. We demonstrated the potential of Federated Learning (FL) in healthcare by experimenting with a public COVID-19 dataset distributed across five artificial data silos. We also presented open challenges that still need to be solved in this domain. For instance, the Non-IID property of the radiographs resulted in higher divergence between model updates, which reduced the training speed and prediction power of the federated model. However, when compared to a purely centrally trained model, our experiments showed that the proposed federated learning strategy still achieved comparatively high model quality as long as sufficient data silos with both class label representations existed, where it clearly outperformed locally trained models. FL therefore enables new ways of collaborative data-driven analytics between medical institutes and organizations, which was otherwise not possible due to location, access, and privacy issues. Such collaborations can contribute, e.g., to a reliable COVID-19 detection and accelerate the diagnostic process in medicine. We plan to take a closer look at federated optimization in the presence of imbalanced Non-IID data and further analyze the impact of the number of silos on model quality. Another interesting direction for future research is to investigate the interpretability and reliability of federated models, which should increase the adoption of FL in data-critical organizations. In addition, to ensure differential privacy in other model architectures, modifications may be needed there as well, which requires further research.

## Data Availability

Data used in this study is openly available and free for research [37]
